# Advancing Research To Address The Health Impacts Of Structural Racism In US Immigration Prisons

**DOI:** 10.1377/hlthaff.2023.00479

**Published:** 2023-10

**Authors:** Chanelle Diaz, Joseph Nwadiuko, Altaf Saadi, Caitlin Patler

**Affiliations:** NewYork-Presbyterian/Columbia University Irving Medical Center, New York, New York.; University of California Los Angeles, Los Angeles, California.; Massachusetts General Hospital and Harvard University, Boston, Massachusetts.; University of California Berkeley, Berkeley, California.

## Abstract

The US is the world leader in imprisoning immigrants. Its mass immigration detention system emerged as an extension of mass incarceration, rooted in a legacy of racist US immigration and criminal laws. Immigration policy is a structural determinant of health that negatively affects the health of imprisoned immigrants, their families, and their communities. The systemic harms of “detention facilities,” which we refer to as “immigration prisons,” have been extensively documented, yet incrementalist reforms have failed to result in improved outcomes for immigrants. We argue that ending the practice of immigrant imprisonment is the most effective solution to mitigating its harms. Community-based programs are safer and less expensive than imprisonment, while also being effective at ensuring compliance with government requirements. We identify several priorities for researchers and policy makers to tackle the health inequities resulting from this structurally racist system. These include applying a critical, intersectional lens to studying the policies and practices that drive imprisonment, engaging affected communities in research and policy development, and creating an accountable and transparent system of data collection and release to inform health interventions. The reliance of the US on immigrant imprisonment is a policy choice with immense social and economic costs; dismantling it is critical to advancing health equity.

The US is a world leader in immigration imprisonment, at an annual cost of approximately $2 billion: As of July 2023 there were more than 30,000 people in Immigration and Customs Enforcement (ICE) custody on any given day, with more than 90 percent of them being held in for-profit immigrant prisons.^1–3^ The growth of the US immigration prison system is an extension of mass incarceration,^4^ rooted in a legacy of racist US immigration and criminal laws, with Black and Latinx immigrants over-whelmingly bearing the harms.^5,6^

People in immigration prisons may be economic migrants, refugees, or asylum seekers, as well as authorized and unauthorized immigrants who have lived in the US for decades. They are detained while awaiting an adjudication of their immigration case or deportation. They have presumably violated immigration law, which is not a crime but a civil violation for which they must go through a process in immigration courts to determine whether they can remain in the US. Border policies such as Title 42 have made it increasingly difficult to request asylum at US ports of entry, forcing many to seek refugee protection after crossing the southern US border, risking detention under ICE’s current priorities for enforcement.^7^

Although these facilities are commonly called “detention facilities,” we refer to them as “immigration prisons” because of their physical characteristics and because immigrants experience them as imprisonment. Immigration imprisonment falls under the purview of civil law,^8^ which means that noncitizens in deportation proceedings can be imprisoned mandatorily and indefinitely, without constitutional due process protections such as the right to appointed counsel.^9^ Since 2009, congressional appropriations bills have required the Department of Homeland Security (DHS) to maintain “not less than 34,000 detention beds” at all times. This has raised concerns about financial incentives, as private for-profit companies—whose lobbying expenditures include those spent lobbying members of Congress who are on the House Appropriations Committee—profit from the growing immigration detention operations needed to maintain this bed quota.^10^

The choice of the US to rely extensively on immigration imprisonment is a manifestation of structural racism that reinforces economic and health inequities. In this commentary we provide the historical context of mass immigration imprisonment as a racist tool of social control and offer a framework for understanding its multilevel health consequences as structural determinants of health. We end our analysis with suggestions for health and health policy research to address the health inequities that result from the current system through an abolitionist lens.

## Structural Racism And US Immigration Prisons

US immigration laws emerged from the nation’s project as a White settler state, with their origins dating to the Naturalization Act of 1790, which limited US citizenship to White people. This set a precedent for the discretionary power of the government to bestow racialized citizenship status.^8^ This precedent was further reified by laws in the 1800s that excluded free Black immigrants from citizenship and by the Page Act of 1875 and the Chinese Exclusion Act of 1882, which excluded most Chinese migration.^4^ The first immigration prisons began in response to this racist legislation.

In 1892 Ellis Island opened as both a welcoming center and a detention facility predominantly for European immigrants. In 1910 Angel Island opened as a West Coast counterpart to Ellis Island that became known for its discriminatory treatment of Asian immigrants. Enforcement of the Chinese Exclusion Act led to disparities in how detention was employed. Although only 10 percent of immigrants arriving through Ellis Island were detained, 60 percent of immigrants at Angel Island were detained.^4^ In both settings, infectious diseases were used as grounds for deportation, as racialized concepts of disease converted medical screenings into tools to measure immigrants’ proximity to Whiteness.^11^ In 1940 the Angel Island facility burned down in an electrical fire, and in 1954 Ellis Island was shut down, ending the government’s immigration prison system for the next two decades.

Immigration imprisonment was formally reinstituted in 1980 with the arrival of Cuban refugees from the Mariel Boatlift and Haitian refugees fleeing the Duvalier regime.^12^ The Carter administration responded to the influx of refugees by converting a nuclear missile base outside Miami into an immigration prison, which is still operational.^12^ Once again, public health concerns fueled the exclusion of racialized immigrants. This included a 1987 ban on immigrants with HIV and the designation of Haitian people as a high-risk group by the Centers for Disease Control and Prevention.^12^ Between 1991 and 1992, tens of thousands of Cuban and Haitian refugees were imprisoned in Guantanamo Bay.^12^ However, there were stark differences between the treatment of Cuban and Haitian refugees. For example, until 2017, Cuban refugees arriving by boat without a visa could pursue permanent residency upon landing on US soil; during the same period, Haitian refugees arriving in the same way could be arrested and deported.^12^

Immigration prisons expanded substantially in the 1980s and 1990s with the arrival of the “War on Drugs.”^13^ The Antiterrorism and Effective Death Penalty Act of 1996 and the Illegal Immigration Reform and Immigrant Responsibility Act of 1996 made immigration imprisonment mandatory for immigrants who committed even minor offenses, creating a pipeline from the criminal system to the immigration system.^4^ The subsequent formation of DHS and ICE in 2003 and several policies mandating police-ICE collaboration facilitated expansion of the immigration prison system.^4^

Latinx and Black immigrants have been disproportionately targeted by immigration enforcement through a process of “racialized illegality.” The enmeshment of immigration enforcement with racist criminal law enforcement, combined with stereotypes and political discourses associating certain immigrants with “illegality,” has contributed to these dynamics.^14,15^ Latin American immigrants make up about 44 percent of immigrants living in the US^16^ but accounted for 94 percent of all detained immigrants in 2015, the latest and only fiscal year for which individual-level data are available.^17^ However, given unequal targeting of Black people by law enforcement,^18^ it is unsurprising that non-Latinx Black immigrants are also disproportionately imprisoned.^6^ In immigration prisons, anti-Black racism persists: Black detained people receive higher bond amounts, are disproportionately punished with solitary confinement, and are more likely to be deported.^6,19^ The disproportionate imprisonment of Black and Latinx immigrants has significant public health implications.

## The Multilevel Health Impacts Of Mass Immigration Imprisonment

### IMMIGRATION IMPRISONMENT AS A STRUCTURAL DETERMINANT OF HEALTH

Structural determinants of health are the socioeconomic and political mechanisms that drive the distribution of power and resources across the population.^20^ In so doing, they shape social determinants such as housing, transportation, and neighborhood safety that affect health outcomes. Immigration policy is a critical but often overlooked structural determinant that worsens health inequities in immigrant communities.^9^ Federal laws dictate immigration status and detention and deportation policy, whereas state and local policies and practices can increase a person’s chances of encountering ICE. These policies and practices in turn drive health equities through several mechanisms, including stress mediated by structural racism, restricting access to social and health institutions, and depriving communities of the material conditions needed to survive.^21^ We review here the existing research on the direct and spillover harms of immigration prisons to the physical and mental health of individuals, households, and communities.

### INDIVIDUALS

Courts have upheld that the government must ensure the well-being and safety of people in their custody. However, oversight of health services for immigrants in ICE custody is governed by inconsistent standards that lack legal enforcement. The provision of care is fragmented; most people receive medical care from employees of for-profit health staffing vendors. A small proportion of immigrants in ICE custody receive care from the ICE Health Service Corps, which is part of the Public Health Service.^9^ Medical care in immigration prisons is focused on acute care, often to the neglect of chronic disease management and preventive care, despite immigrants being subject to prolonged detention.^22^

Immigration imprisonment can make even healthy people sick, through an accumulation of physical and mental trauma. While detained, people are subjected to the neglect of basic needs, such as nutrition, and are placed in a physical environment that accelerates illness through overcrowding, poor sanitation, and lack of recreation.^23^ Physical violence and sexual assault are prevalent in immigration prisons,^24^ with sexual and gender minorities at increased risk.^25^ In addition, people experience emotional distress related to dehumanization, perceived injustice, and isolation while facing systemic barriers in accessing high-quality health care.^23^ These conditions cumulatively worsen health outcomes among imprisoned immigrants.^26^

Existing research demonstrates that immigration imprisonment leads to a deterioration in mental health. Multiple stressors at each stage of migration, including physical and structural violence, fear, poverty, and discrimination, can contribute to worsening mental health status among immigrants.^27^ These stressors are acutely exacerbated during imprisonment. Duration of imprisonment is associated with worsening symptoms of anxiety, depression, and posttraumatic stress disorder.^28^ Prolonged detention and increases in the use of solitary confinement in immigration prisons, including among people with serious mental illness, also have significant negative health implications.^19,29–31^ A recent analysis of hospitalizations among people in ICE and Customs and Border Protection custody in Texas and Louisiana found a high burden of admissions for psychiatric illnesses, especially suicidal ideation and self-harm.^32^ The proportion of deaths attributed to suicide in immigration prisons has increased dramatically. In 2020 the rate of suicide among people detained by ICE was eleven times the suicide rate of the previous decade.^33,34^ In contrast, release from immigration prisons is associated with decreased psychological and physical stress.^35^

The COVID-19 pandemic underscored the harms of immigration prisons. Alarmingly, death rates in immigration prisons accelerated sevenfold between 2019 and 2020, even as the average population in custody decreased by a third during the early pandemic.^33^ Several systemic issues contribute to deaths in detention even outside the context of the pandemic, including delivery of grossly substandard health care, lack of patient-centered care, bias and discrimination, language injustice, and other structural barriers that deprioritize a person’s health.^36^ The COVID-19 pandemic amplified these risks.^37^

### HOUSEHOLDS

Immigration prisons also exert spillover health harms onto children and adults from the households of detained people. Families bear the collateral costs of imprisonment, which removes millions of dollars from local communitiesinlostwages.^1^ Family members are forced into a state of collective liminality, in which they experience “heightened threat and uncertainty” as they await their loved one’s (temporary or permanent) release into the US or deportation.^38,39^ One study described how “suddenly single mothers” whose husbands were detained experienced extensive stress, anxiety, fear, and worry.^40^ Another study found that family members of imprisoned immigrants began avoiding key social institutions and health-promoting government benefits to which they were entitled, to avoid additional exposure to government officials.^41^

Children of imprisoned immigrants also have heightened psychological distress, which often extends for years after the initial parental detention.^42,43^ The compounded vulnerability faced by children with detained parents can result in decreased engagement in school due to stigma and the fear of exposing family members to immigration authorities.^41^

### COMMUNITIES

Growing evidence suggests that immigration prisons, and the immigration enforcement practices that fill them, harm communities. Fears related to immigration enforcement have been associated with population-level reductions in Medicaid and Special Supplemental Nutrition Program for Women, Infants, and Children enrollment; delays in receiving prenatal care; increases in low birthweight among infants born to Latinx mothers; and increased childhood poverty.^44^ This likely relates to the spillover impacts of “racialized illegality,” which refers to racialized groups, such as Latinx people, being targets of stereotypes that associate them with illegality even if they have authorized status.^14,45^ Some people also worry about immigration enforcement because they belong to mixed-immigration-status families (whose members include people with different citizenship or immigration statuses). For example, in one study involving a preventive health intervention for Latinas in Southern California, participants who resided near an immigrant prison, regardless of their immigration status, benefited less from the intervention, reported higher anxiety levels and decreased mobility around their neighborhoods, and requested resources to respond to ICE surveillance of their communities.^46^

The COVID-19 pandemic further underscored the interconnectedness of immigrant prisons and the communities surrounding them. Systemic failures by ICE to mitigate COVID-19 transmission early in the pandemic, including inadequate access to personal protective equipment and basic sanitation, led to disproportionate cases and deaths in immigration prisons^33,37,47^ and heightened community spread.^48^ There is an extensive body of literature describing other aspects of the immigration prison system that have implications for population health, including border militarization,^49^ antiimmigrant rhetoric,^50^ and the economic impacts of immigration prison building.^51^

## Moving Toward Abolitionist Policy Solutions

Policy solutions to immigration prisons can be broadly categorized as abolitionist or incrementalist, mirroring the dynamics in broader efforts to mitigate the US mass incarceration crisis.^52^ Abolitionist approaches call for ending immigration imprisonment, as well as other carceral technologies used in immigration enforcement, such as electronic monitoring. Incrementalist or reformist approaches have focused on improving conditions in immigration prisons, including health resources, and protecting vulnerable people such as pregnant women and children.

Incrementalist approaches may unintentionally reinforce this structurally racist system. For example, calls for improved health care within immigration prisons are often met by increased funding for ICE but do not result in improved outcomes for immigrants.^53^ Indeed, DHS and private for-profit prisons fail to disclose systematic data on the clinical characteristics or outcomes of people in their custody. Instead, the public has relied on scholars, advocates, and government watchdog organizations, who have long documented alarming inadequacies in health care and conditions of confinement.^53–56^ A recent review of deaths in immigrant prisons identified violations of ICE’s own medical standards in 78 percent of cases.^57^ These are not reasons to abandon calls to improve the safety and quality of medical care; however, reform should not distract from the deeply systemic harms.

Academic and legal scholars, medical and public health professionals, and immigrants’ rights advocates have called for the release of detained people as the most effective solution to mitigating the numerous harms incurred by immigration imprisonment.^13,58^ Furthermore, there is little empirical support for using immigration imprisonment to ensure compliance with immigration legal proceedings. Evidence shows high levels of compliance from immigrants regardless of histories of immigration imprisonment.^59^ In recent years there has been a vast and troubling expansion of electronic surveillance in place of imprisonment, and although its health implications remain understudied, recent qualitative research suggests that immigrants experience itasharmful.^60^ Still, pilot programs testing alternatives to imprisonment in several countries, including the US, have been shown to be cost saving and effective at ensuring court appearances.^61^ One example is the Community Support Project, a pilot program in the United Kingdom that serves detained migrants facing deportation who have been convicted of criminal offenses. Detained people are connected to case workers, who work with them to develop a transition plan and navigate needed services in the community, including mental health and legal support; 93 percent of enrollees have not reoffended.^62^

Recent literature has demonstrated the positive health impacts of release on imprisoned people. A two-wave panel study of seventy-nine immigrants who were detained and then later released in California revealed that levels of psychological and physical stress symptoms decreased by nearly a third, and the probability of excellent general health increased by nearly two-thirds, after release.^35^ The study identified reunification with family, physical freedom, and autonomy as potential mechanisms for improved health.

Successful abolitionist praxis is achievable and will require engagement at all levels of government to dismantle the local, state, and federal scaffolding that maintains immigration prisons. It is also important to acknowledge the overlapping sociopolitical and economic drivers of mass incarceration and mass immigration imprisonment. As just one example of this interconnectedness, after the Biden administration’s 2021 executive order to phase out Department of Justice contracts with privately operated prisons, those same private prison beds were quickly filled with immigrants.^3^ Researchers and policy makers should engage with affected communities to consider creative approaches to closing immigration prisons. For example, county-level organizing campaigns across the country have led local officials to end contracts with ICE and sheriffs’ offices to cancel their contracts to deliver immigrant arrestees to ICE custody.^63^

## Advancing Research And Policy To Address The Health Impacts Of Mass Immigration Imprisonment

Building on Maria-Elena De Trinidad Young and Steven Wallace’s proposed research agenda to improve immigrant health,^64^ we identify several priorities for health and health policy research that can begin to tackle the health inequities resulting from immigration imprisonment.

First, research that goes beyond identifying the health consequences of structurally racist systems such as immigration prisons is critically needed to inform inclusive immigration policy. This requires a critical, intersectional lens on the inequitable systems and structures that drive vulnerability as the unit of analysis.^65^ Multi-disciplinary teams have already created tools to measure the effects of structural racism and xenophobia.^66,67^ Policy implementation science can inform strategies to implement alternatives to immigration prisons while examining their population health impacts.

Second, immigrant communities affected by imprisonment, in addition to front-line practitioners and advocates, should be centered in research and policy analysis. This will ensure that researchers and policy makers emphasize the needs and priorities of the community in developing creative solutions for just transitions from imprisonment.^68^

Third, assumptions that are embedded in research funding and policy about whose health deserves attention should be dismantled.^69^ These social categorizations can reinforce exclusionary policies and uphold structural racism.

Fourth, a strengths-and-assets-based approach to studying the societal benefits of ending the practice of immigration imprisonment should be employed. Research that uses narratives to humanize people in immigration prisons and highlights the strengths of immigrant social movements can influence cultural change and, eventually, policy.

Fifth, an accountable and transparent system of data collection and release to understand the long-term health impacts of immigration imprisonment and inform intervention development should be created. Community- and health system–based interventions that are trauma informed and meet the needs of individuals and their households after imprisonment are urgently needed.

## Conclusion

In a prior issue of *Health Affairs*, Paula Braveman and colleagues argued that “systemic racism is so embedded in systems that it often is assumed to reflect the natural, inevitable order of things.”^70^ We argue that the extensive reliance of the US on imprisonment in a system of civil law is a policy choice rooted in structural racism. In the case of immigration imprisonment, racism defines whose humanity it is acceptable to ignore, at immense cost to the health of the individual, the household, and the broader community. Dismantling this harmful system is imperative to addressing inequities in immigrant health. ▪
